# Influence of Design Parameters on Fresh Properties of Self-Compacting Concrete with Recycled Aggregate—A Review

**DOI:** 10.3390/ma13245749

**Published:** 2020-12-16

**Authors:** Rebeca Martínez-García, P. Jagadesh, Fernando J. Fraile-Fernández, Julia M. Morán-del Pozo, Andrés Juan-Valdés

**Affiliations:** 1 Department of Mining Technology, Topography, and Structures, University of León, Campus de Vegazana s/n, 24071 León, Spain; fjfraf@unileon.es; 2 Department of Civil Engineering, Coimbatore Institute of Technology, Coimbatore 641014, Tamil Nadu, India; jaga.86@gmail.com; 3 Department of Agricultural Engineering and Sciences, University of León, Avenida de Portugal 41, 24071 Léon, Spain; julia.moran@unileon.es (J.M.M.-d.P.); andres.juan@unileon.es (A.J.-V.)

**Keywords:** recycled aggregates, self-compacting concrete, design parameters, fresh concrete properties, mix design

## Abstract

This article presents an overview of the bibliographic picture of the design parameter’s influence on the mix proportion of self-compacting concrete with recycled aggregate. Design parameters like water-cement ratio, water to paste ratio, and percentage of superplasticizers are considered in this review. Standardization and recent research on the usage of recycled aggregates in self-compacting concrete (SCC) exploit its significance in the construction sector. The usage of recycled aggregate not only resolves the negative impacts on the environment but also prevents the usage of natural resources. Furthermore, it is necessary to understand the recycled aggregate property’s role in a mixed design and SCC properties. Design parameters are not only influenced by a mix design but also play a key role in SCC’s fresh properties. Hence, in this overview, properties of SCC ingredients, calculation of design parameters in mix design, the effect of design parameters on fresh concrete properties, and the evolution of fresh concrete properties are studied.

## 1. Introduction

In recent decades, the construction industry has experienced exponential growth worldwide and throughout the territory of the European Union (EU). This growth has caused a very important increase in the generation (among others) of so-called construction and demolition waste (C&DW). The developed world, or first world, is the great consumer of raw materials on the planet and also the largest generator of waste. According to data from the European Statistical Office, Eurostat, each EU citizen produces an average of 2000 kg of waste per year, without counting waste from mining (including the latter, the figure would exceed 5000 kg/person/year) [1]. Of this set of waste, more than a third corresponds to the construction sector.

After water, aggregates (basically sand and gravel) are the raw material most consumed by human beings and their main use is a key part of construction materials such as concrete, mortar, bituminous mixtures, etc. It is necessary to promote the recovery of waste generated by these activities, thus reducing the extraction of natural aggregates and the efficient means of waste management [2,3]. Concrete is the world’s second most consumed material by human beings next to water. Due to the modernization of the world, the demolition of concrete is increasing day by day [4]. Hence, usage of such demolished waste is more focused by researchers across the globe and even several countries like Italy and Denmark are making standards and principles regarding it. Few countries (like Denmark and Germany) accomplish reuse percentages of over 80% [1] but several other countries across the globe have reuses percentage below 10% [5], so reuses problems remain the same. Usage of recycled aggregate (RA) not only provides the solution for landfill deposition but also preserves natural resources for natural aggregate quarrying.

**Figure 1:**
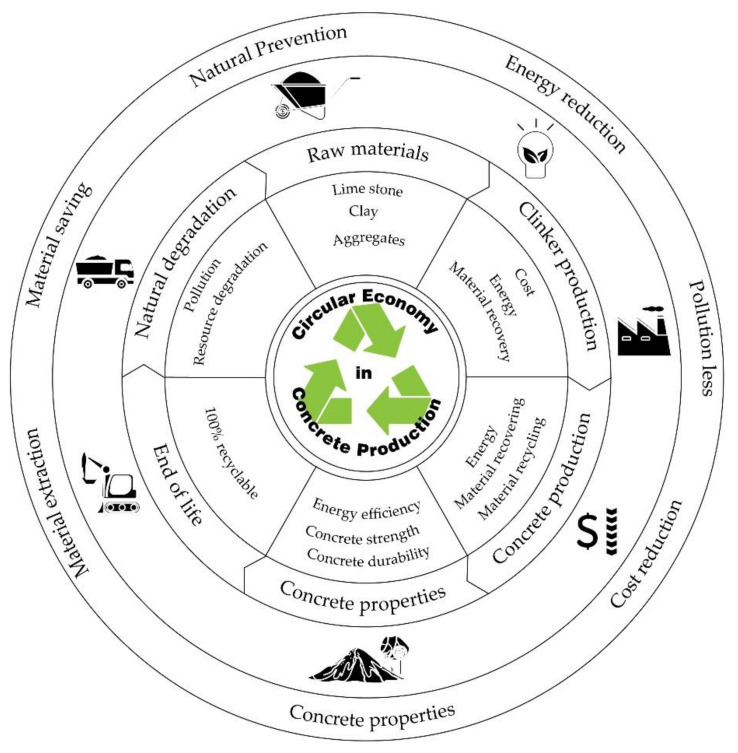
Concept of circular economy.

Apart from this, it also reduces the overall footprint of concrete by a reduction in carbon dioxide (CO_2_) emission [6,7], and energy associated with it. Usage of waste materials leads to a circular economy [8] which will respond to environmental challenges as shown in Figure 1. Usage of RA in concrete leads to approximately a 20% drop in CO_2_ emissions and 60% preservation of natural resources due to aggregate quarrying [9]. Most industry across the globe undergoes a sustainable process, likewise the construction industry [10]. Over the past few decades, there have been a lot of changes occurring in the concrete sector to traditional ways of its production, notably the addition of minerals, chemical admixture, new supplementary cementitious materials (SCM), etc. To accomplish the sustainability in the concrete production process, the following measures are to be taken:

- Reduction in carbon dioxide emission produced during raw material quarrying and processing [11,12];
- Reduction in natural resource utilization [12,13];
- Reduction in cost [14,15];
- Enhancement of fresh and hardened properties of concrete [9,16,17];
- Less energy consumption during the production process [18];
- Reduction in noise level due to vibration caused by compaction of concrete [14].

Today, local and state administrations, the scientific community, and the general population are increasingly aware of the depletion of natural resources and advocate for sustainable development. As a result of this development, the need arises to recycle waste, where resources are part of a circular economy and can be sustained from generation to generation as depicted in Figure 1. Society as a whole has become conscious of the need to combine economic development with sustainability and protection of the environment. It is in this context where the need arises to study new applications and developments for waste from C&DW. Obtaining cement-based materials made with RA (obtained from the treatment of C&DWs) has been one of the most studied applications. Although, indeed, there are currently some reliable studies that support the use of C&DW in conventional concrete [16,17,18,19,20,21,22], in recent years some studies on the use of RA have begun to be published with self-compacting concrete (SCC), although they are still rare [23,24,25,26,27,28,29,30,31,32,33,34,35,36,37,38,39]. Most of these studies compare the various fresh and hardened properties of SCC with RA and without RA (natural aggregates), apart from SCC mix design. Most of the studies only substitute the coarse fraction of the aggregate, but there are some (the fewest) that also substitute the fine fraction [27,29,30]. Numerous tests have concluded that the incorporation of RA into concrete as alternatives for natural aggregates in small proportions (≤20%) does not cause a reduction in the performance of these recycled concretes. These studies have made it possible for these types of recycled materials to be included in regulations and/or recommendations all over the world. In general, the guidelines for its use are usually the following: limitation of the recycled coarse aggregate (RCA) content to 20 wt.% on the total coarse aggregate content, opening the possibility of experimental studies for larger substitutions; the use of recycled fine aggregates (RFA) is not usually allowed, regardless of their nature, although some studies [27,29,30] show that substitutions of up to 10% do not produce significant variations in concrete characteristics; the use of mixed RA is excluded (that is, with the presence of ceramic materials in percentages by weight of around 30% or higher) [38]. Regulations and codes usually limit the composition and physical and mechanical characteristics of recycled aggregates for concrete for structural use. For example, in Europe, the UNE-EN 12,320 [40] and UNE-EN 933 [41] standards limit the content of ceramic materials to 5%, the gypsum content to 1%, the absorption capacity of aggregates below 5%, or the Flakiness index below 35% among others. These limits may vary from country to country.

However, the fact that international standards do not include the possibility of using mixed RA, the limitation on the total content substituted, and the impossibility of using fine fraction aggregates from CD&W, make it a challenge for the scientific community to develop studies and techniques capable of achieving these objectives, increasing the reuse capacity of these aggregates and promoting their use and recovery.

One of the advantages of SCC over conventional concrete is the handling process and the problem associated with it. One of the possible solutions to achieve the measure stated above is the fresh concrete properties of SCC with RA. Fresh properties of SCC play a key role in the behavior of hardened properties [42]. SCC composition is characterized by a high content of cement and fines, a reduction in the proportion of coarse aggregates, and the use of next-generation additives [43]. Due to the self-flowing ability of SCC, it requires a greater powder content, which leads to an increase in cost [44]. The presence of Ordinary Portland cement in a higher quantity of mix not only increases the cost but also liberates the high heat of hydration that is associated with concrete cracking [45]. To reduce the cost and heat of hydration of SCC, several investigators have introduced the usage of mineral admixtures [14,23] and the usage of RA [14]. In addition, the maximum volume of occupation in concrete is an aggregate that will occupy 60% to 70% [46] depending upon usage. Hence, any small change in the aggregate has a significant effect not only on the concrete properties but also on the cost. Hence, several researchers have examined the use of RA as a replacement for fine and coarse aggregate [14] in recent years. For the past two decades, extensive studies have been carried out on the effective usage of RA [32].

Ingredients used for SCC are similar to that of conventional concrete but include the addition of additives such as superplasticizers (SPs) (or viscosity modifiers) that are essential to avoid segregation and exudation of the mixture. This type of concrete requires special attention regarding the choice of materials, guaranteeing their uniformity and consistency, which makes them more complex when it comes to being dosed with substitute RA, due to their initially possible heterogeneity [43]. Several studies confirm the greater absorption capacity of RA, around 10% extra water, a premise to take into account. This higher water demand can be offset by the use of an SP [33,47,48,49,50,51]. Other authors also use other valid techniques such as pre-saturating the aggregates [52].

Adhered mortar on RA had a greater influence on the properties of RA [53] apart from the crushing type used [54]. The crushing type of aggregate determines the shape and size of the recycled product produced [54]. There is a possibility of four interfacial transition zone (ITZ) found in RA concrete: 1. ITZ (formed due to hydration reaction occurring between RA and new mortar) [55]; 2. ITZ (formed due to the reaction occurring between new aggregate and new mortar) [55]; 3. ITZ (formed due to reaction between new aggregate and old mortar) [56]; and 4. ITZ (formed due to reaction between old mortar and RA). The development of ITZ from old mortar and RA is very slow. And already-formed old ITZ by old mortars has microcracks and voids in it, which is responsible for a reduction in the hardened properties of RA concrete [23,56]. Properties of RA depend upon the source from which it is extracted [56] and has a direct influence on the RA concrete properties. Several researchers observed that the RA resulting from high strength concrete can be used as a 100% replacement for natural aggregate for production of RA concrete [4,57,58]. One of the main disadvantages in using RA as a commercialization of the product is due to the presence of this excess mortar adhering to it. But few investigations on SCC incorporated with RCA show mechanical and durable properties similar to that of the SCC with NCA by increasing cement content and SCMs, adjusting water to the cement (W/C) ratio and the size of RCA [59,60,61,62,63].

Quantity of RFA generated from C&DW occupies 20 to 50% of total C&DW [63]. Even several authors [64,65] recommended using a grinding technique to convert this fine part of fine aggregate to reactive powder (RP). RP consists of reactive (silicon dioxide, calcium hydroxide (CH) and calcium silicate hydrate) materials making it a higher pozzolanic activity. The reactive form of minerals is confirmed with help of microscope studies [66]. Even the surface area of RP particles is found to be higher than the cement [66], with an average particle size of 0.0154 mm. Usage of RP in SCC increases the strength performance due to the possible accessibility of extra pozzolanic material in RP and the possibility of a reaction with unreacted CH [66]. Due to the shape and texture of RP, it possesses better characteristics than other pozzolanic materials, which further enhance the bonding and interlocking of the cement matrix [66]. RP also occupies the pores in the cement matrix and provides better packing density due to its fine particle size.

The use of RA in the production of SCC has been gradually increasing due to its economic and environmental advantages derived from the fusion of the two techniques. On the one hand, the advantages of SCC: optimal compaction without vibrating, fluidity, complex and specially reinforced works, increased quality of finish in exposed concrete, greater adherence, etc., and on the other the advantages of RA: less demand for extraction of new quarry aggregates, reduction of pollution produced by C&DW, savings in transport costs, low energy consumption, reduction of investments since a good quality material is produced at a low cost, resources are optimized, etc. However, information on the quality of SCCs with RCA is still scarce. The quality of SCC depends on design parameters that influence the properties of concrete. Some of the design parameters considered in these studies are W/C ratio, water to powder (W/P) ratio, the ratio of coarse aggregate to total aggregate, percentage of powder content in total volume, and weight of SP in terms of percentage of cement. The preliminary objective of this article is to increase the usage of waste materials in the construction industry, especially SCC in order to understand the effect of design parameters of SCC mix on the fresh concrete properties. This study provides very useful information on design parameters, the influence of design parameters on mix design and fresh concrete properties, and comparison with available standards, which are helpful for practical use in concrete production.

**Figure 2:**
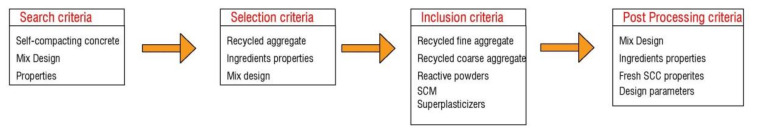
Flow diagram for selection criteria.

Figure 2 shows the search criteria, the selection criteria, and the subsequent processing of the articles studied in this review.

## 2. Material Properties of SCC Ingredients

The origin of SCC can be diverse, but there is a definite role of ingredient properties on the SCC fresh concrete properties. Hence, it is necessary to understand the properties of ingredients used in SCC. Tuyan et al. [14] used a laboratory-made aggregate from concrete waste, crushed with a jaw crusher. Pereira et al. [32] used two types of RA from a C&DW recycling plant. Señas et al. [49] used an RA from construction sites. The material was crushed with a jaw crusher. It was sieved to achieve a size similar to that of NCA and washed before adding it to the SCC. Duan et al. [67] used an RA from a C&DW recycling plant. The fine fraction was obtained by further grinding the RCA. Tang et al. [68] used an RA of unwashed, crushed concrete from a C&DW waste recycling plant. The aggregates were immersed in water 24 h before and air-dried for 1 h before adding them to the concrete.

In Djelloul et al. [69], the RA were obtained by crushing 1 m × 1 m concrete slabs with a thickness of 10 cm, manufactured in the laboratory, and kept for 28 days in water. They were first subjected to manual crushing and later with a mechanical crusher. Sasanipour et al. [56] used laboratory crushed concrete aggregates. They were first crushed with a small machine to produce RCA and then manually crushed using a hammer to produce the RFA. Aslani et al. [70] used aggregates that came from the demolition of buildings and concrete infrastructures. The rubber aggregates were obtained from the mechanical grinding of tires at the end of their useful life. Ouldkhaoua et al. [45] used metakaolin as obtained from the calcination of kaolin. Nieto [71] used an aggregate from crushed structural concrete. Dapena [72] used RA from structural concrete, crushing plants with a strength between 15 and 35 MPa.

The average value of ingredients used for SCC other than RA by various researchers is tabulated in Table 1. It is observed that the average specific gravity is obtained as 3.108. Specific gravity for fine and coarse aggregate investigated by several authors are 2.651 and 2.646. From Table 1 and Table 2, it is observed that the physical properties of natural aggregate are higher than that of the RA due to old mortar adhering to it. The specific gravity of RA is lower than that of natural aggregate and is observed by most researchers, this leads to a density of RCA mixture as it it lower than that of the natural aggregate mixture. Higher water absorption of RA is observed from Table 2 when compared to Table 1 and it is reported by most authors.

RA size investigated by most researchers less than 16 mm is observed in Table 2. It is also observed that the specific gravity and bulk density are lesser than natural aggregate. And also, higher water absorption than natural aggregate with an inconsistent form of fineness modulus of RA is noted. The quality and quantity of cement paste adhering to the surface RA affects the properties of it [15,32]. Accordingly, the RA has a lower density, higher water absorption, and lower mechanical strength properties [15]. From Table 2 and in past studies it can be observed that the density of RA is lower than that of natural aggregate due to the coating of cement paste on the surface of RA. This excess amount of cement paste results in an increase in the water absorption capacity of RA [15,32]. Coarse aggregate particle size is largely a result of the adhering of excess cement paste on it when compared to fine aggregate. Hence, most researchers have used an RCA size less than 16 mm. Coarser RCA shows a more negative effect than finer RFA [77,78,79]. To achieve the same workability compared to control concrete, concrete with RA requires a greater quantity of water due to the presence of excess cement paste [78]. This result in a higher water to binder ratio leads to the formation of more porosity in RA concrete and reduction in durability properties. Hence, usage of mineral admixtures may reduce this effect, since they provide the same workability to the mixture without increasing the water to binder ratio [15].

Salesa et al. [6] observed that the introduction of RCA in SCC leads to drops in fresh concrete properties [6]. This drop in fresh concrete property is due to two factors influenced by RCA. Firstly, RCA has a greater surface roughness to react and secondly, RCA has more fine particles adhering to the surface than natural aggregates and because of this they required more water to achieve similar fresh concrete properties.

Zhang et al., 2016 [80] observed that old ITZ in RCA did not develop with an increase in curing time, irrespective of surface treatment on RCA using nanomaterials (nano silica + nano calcium slurries or cement + nano silica slurries). Nanomaterials are not able to infiltrate into old ITZ to react with a few unhydrated particles present in it. New ITZs formed in RCA (surface treated by nano materials) were enhanced significantly. ITZ improvement is directly related to the elastic modulus of that particular ITZ. Old mortar adhering to RCA was surface-strengthened by nano surface treatment, resulting in enhanced RCA properties (water absorption, crushing value apparent density).

The higher water absorption nature of RCA results from the higher absorption of cement mortar attached to aggregate particles [81]. Cui et al., 2015 [81] further found that RCA with a higher water absorption nature can impact the fresh and hardened properties of concrete. Higher water absorption of RCA is due to the porous nature of old paste and the cracks present in it. RCA with surface modifications using an alkaline organosilicon modifier was effective in reducing the initial water absorption and it does not alter the mechanical properties of concrete when used in it.

Kou and Poon, 2010 [82] found that there are enhanced properties of RCA that can be observed when RCA is impregnated with Polyvinyal alcohol (PVA). There were improved mechanical and durability properties of concrete with RCA impregnated with PVA.

The enhancement of RA properties is observed after treatment when compared to untreated RA [15]. Enhanced properties are decreased in water absorption and an increase in the specific gravity of treated RA. Treated aggregate shows an improvement in fresh and hardened properties when compared to untreated aggregates.

As stated earlier, several works have been carried out to enhance the properties of RCA. Two methods are adopted in the literature either to remove the adhered mortar or to improve the adhered mortar quality [23,83]. In the first method, RCA is pre-soaked in different types of acids like hydrochloric acid and sulphuric acid at different concentrations to remove adhered mortar [84]. For the second method, a surface treatment like coating is carried out to improve the quality of RCA without removing the old mortar adhering to it. Surface treatment materials like microbial carbonate [85], silane-based water repellent [86], PVA [82], alkaline organosilicon modifier [81], silicate-based solution [87], and pozzolanic materials [88] have been used with different concentrations to improve the concrete properties.

## 3. Mix Design

The development of the SCC reference mix based on the strength achieved at 28 days as the average value of authors are shown in Table 3. Dosages of ingredients without RA as proposed by several authors are compiled for the production of control SCC in order to identify the range of ingredients required for it. It is also observed that the unit weight of the mix is found to be less than the standard weight of concrete. The proportion of coarse aggregate to total aggregate from the reference mix is 0.477, which is suitable for SCC as prescribed by subsequent mixes by several authors. Design constants for control mix from Table 3 are water to binder ratio as 0.34, water to powder ratio as 0.26, % of SP as 0.94%, % of SCM as 0.5, and filler to binder ratio as 0.29.

We have selected the most representative studies of the use of SCC concrete with RA published in recent years and summarized in three tables (Table 4, Table 5 and Table 6) based on the weight basis of ingredients.

Tuyan et al., 2014 [14] design four types of mixes with substitutions of 0%, 20%, 40%, and 60% of NCA with RCA. The cement was class C with a content of 315 kg/m^3^. Three W/C ratios are used, 0.62, 0.69, and 0.76. The SP content was adjusted about the percentage of replacement from 0.95% to 1.97% of the cement weight. The water absorption of the RA was 4.80%.

Pereira de Oliveira et al. [32] designed four types of mixes with substitutions of 0%, 20%, 40%, and 100% of NCA with RCA employing two types of RA, using CEM I 42.5-R at 284.9 kg/m^3^. The W/C ratio was 0.56 and 0.57. The SP was adjusted about the replacement ratio from 1.193% to 2.106% of the cement weight. The water absorption of the two used RA was 4.10% and 4.05%.

Señas et al., 2016 [49] design six types of mixes with substitutions of 50% of NCA with RCA and 20% of natural fine aggregate (NFA) with RFA. The cement was Portland type I with a content of 415 kg/m^3^. W/C ratio was used as 0.50. They used two different high range water reducing admixtures as “S” and “H” types. The SP content was adjusted from 0.7% to 1.50% of the cement weight.

Duan et al., 2020 [67] designed nine types of mixes with a combination of RFA and RCA substitution. The C1 series with a 10% substitution of NFA for RFA and substitutions of 25%, 50%, and 100% of NCA for RCA. The C2 series with a 20% substitution of NFA for RFA and substitutions of 25%, 50%, and 100% of NCA for RCA. The cement used is PO425 with a content of 430.5 kg/m^3^. The W/C ratio is constant at 0.57. A constant SP of value 0.25% of the cement weight. The water absorption of the RA was 6.53% and was previously submerged in water for 24 h.

Tang et al., 2020 [68] design five types of mixes with substitutions of 0%, 25%, 50%, 75%, and 100% of NCA with RCA. The cement was ordinary Portland cement, type I, with a content of 445 kg/m^3^. Three W/C ratios used 0.35. The SP content was 0.1 of the cement weight in all mixtures. The water absorption of the RA was 7.75% and was previously submerged in water for 24 h.

Djelloul et al., 2018 [69] design fifteen types of mixes with substitutions of 0%, 25%, 50%, 75% and 100% combined coarse and fine recycle aggregates. The cement was CEM II/A 42.5, with three different contents, 359, 434 and 507 kg/m^3^. The W/C ratio and SP dosage were kept constant at 0.38 and 1.5% by weight of cement, respectively. The water absorption of the RA was 3.21 and 7.39 for RCA and 6.3 for RFA.

Sasanipour et al., 2019 [56] designed twelve types of mixes with substitutions of 0%, 25%, 50%, 75%, and 100% combined RCA and RFA. The cement was Portland cement type II, with 420 kg/m^3^ content kg/m^3^. The W/C ratio was kept constant at 0.4 and SP content was adjusted from 0.9% to 1.15% of the cement weight. The RA was previously submerged in water for 24 h.

Aslani et al., 2018 [70] designed three series with 5 mixes each. Series I with substitutions of 0%, 10%, 20%, 30%, and 40% combined coarse and fine recycle aggregates. Series II crumb rubber replacement is kept constant at 20% of the volume of coarse aggregates, and NFA is then replaced by RFA at 0%, 10%, 20%, 30%, and 40% of fine aggregate volume respectively. Series II kept constant coarse aggregate replacement consisting of 50% scoria lightweight aggregates, NFA is then replaced by RFA at 0%, 10%, 20%, 30%, and 40% of fine aggregate volume, respectively. The cement was type GP, with 450 kg/m^3^ content kg/m^3^. The W/C ratio was kept constant at 0.45 and SP content was adjusted from 1.222% to 1.778% of the cement weight.

Ouldkhaoua et al., 2020 [45] designed nineteen mixes, natural sand has been replaced with recycled cathode ray tube glass (CRTG) and metakaolin at levels of 0%, 10%, 20%, 30%, 40%, and 50% by weight and the cement has been partially replaced by MK at substitution ratios of 5%, 10%, and 15% by weight. The cement was Portland CEMI 42.5, the content ranges from 399.15 kg/m^3^ to 469.59 kg/m^3^. The W/C ratio from 0.4 to 0.47 and SP content was adjusted from 0.8% to 1.1% of the cement weight.

Nieto et al., 2019 [71] prepared five different mixes, each divided into two groups. The first group contained concrete with different water-cement ratios W/C 0.55, 0.50, and 0.45, with different quantities of cement 367, 386, and 408 kg/m^3^, respectively and different substitution rates of NCA with RCA 0%, 20%, 40%, 60%, 80%, and 100%. The cement was CEMI 52.5 N/SR. The SP content was kept constant at 1.5% of the weight of cement. The water absorption of the RA was 5.35%, 5.83%, and 6.61% and was previously submerged in water.

Dapena et al., 2011 [72] designed ten mixes with substitutions of 20%, 50%, and 100% of coarse RA combined with substitutions of 5%, 10%, and 15% of fine RA. Prepared mixes with a constant W/C ratio of 0.50. The cement was type CEMI 42.5 R/SR, with 380 kg/m^3^ content kg/m^3^. The SP ratio was kept constant at 0.70% of the weight of cement. The water absorption of the RA was 4.12%.

To study one of the objectives, the authors grouped the mixed design in the literature into the following four families based on the design parameters.

- Family IA, W/C ratio as a varying parameter and its influence on the ingredients. Family IB, W/C ratio as a constant parameter;
- Family II, W/P ratio has varying parameters and has an influence on the ingredients;
- Family III, the influence of superplasticizer dosage on the ingredients.

The following features were observed from the above Table 7:

- For family IA mix, an increase in W/C ratio shows a decrease in the percentage of cement content in terms of unit weight;
- For the family IA mix, an increase in the W/C ratio causes an increase in the SCM content of the mixes;
- For the family IB mix, an increase in the W/C ratio is inconsistent with cement and SCM content;
- For a family I mix, the filler content varies concerning the W/C ratio but the sequence shows that a greater amount of filler material results in a higher water content of mixes;
- For the family, I mix, increase in the percentage of aggregate in mixes results in an increase in water required to achieve the same workability of the mix

The following features are observed from the above Table 8

- With an increase in W/P ratio, powder content of mix is decreased;
- With an increase in the W/P ratio, the proportion of fine aggregate content is decreased and coarse aggregate content is increased;
- With an increase in the W/P ratio, the proportion of replacement of fine aggregate by RFA is decreased;
- Water and SP content in the mix has an indirect relationship with the total powder content of the mix.

The following features are observed from the above Table 9:

- An increase in the replacement of aggregate by RA results in an increase in SP to achieve the same consistency and is observed in mixed designs in the literature;
- Increases in SP result in an increase in SCM content of the mixture;
- Increase in SP results in an increase in the filler quantity of the mix;
- An increase in SP content increases the total aggregate content of the mixture.

Apart from the above common observations, other works of literature show the most valid features of SCC properties. Most previous research has confirmed that the reduction percentage of recycled concrete elastic modulus is higher than the strength reduction [89,90]. An increase in the flowability of recycled SCC is achieved by adding a greater quantity of admixtures [70]. Due to an increase in SP content and porous nature of RA, it leads to instability and segregation of mix, which was controlled by adding a viscous modifying agent (VMA) [70,91]. The addition of GGBFS in SCC enhances the durability properties of SCC [69]. With an increase in the replacement percentage of NCA by RCA, the fluidity of SCC is reduced [71] due to the higher water absorption characteristics of RCA. This leads to an increase in the utilization of SP in SCC to maintain the same fluidity [71]. From the material and economic optimization perspective, there is a more focus on the reduction of SP quantity in the mix [71]. The addition of marble powder results in a decrease in void content due to its filler effect [75] in the mixture.

## 4. Influence of Design Parameters on Fresh Concrete Properties

For each fresh SCC property and every author, the results/findings obtained are presented in the form of a table/graph and the new tendencies are identified and studied based on the design parameters obtained from the mix design. A comparison of similar kinds of experiments is made from various authors to understand the RCA trends on fresh properties.

### 4.1. Effect of the Water to Cement Ratio on Fresh Concrete Properties

The researchers concluded that several parameters are used to evaluate the slump flow, which is defined as free fluidity and flow in the absence of barriers. The parameters considered are the time required by SCC takes to form a 500 mm circle, called flow time (T_500_), and the slump flow (SF) diameter. SF ratio is defined as the ratio between the slump flow by RA SCC to the slump flow by natural aggregate SCC. A common observation made by most researchers is that increase in the replacement of coarse aggregate by RA results in T_500_ increases with SF decreases.

**Figure 3:**
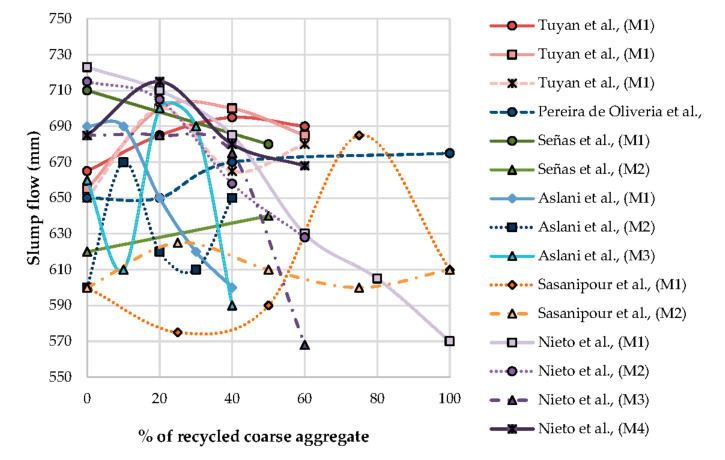
Effect of recycled coarse aggregate on slump Flow and its classification based on EFNARC [95].

EFNARC [92] classified SF into three categories viz. SF1 (550–650 mm), SF2 (660–750 mm), and SF3 (760–850 mm). This means that the slump flow can differ from 550 mm to 850 mm. Table 10 shows that most of the RA SCC shows that most of the literature shows SF1 and SF2, which is also confirmed by Figure 3. EFNARC [92] categorized T500 mm spread flow into two classes, namely VS1 (≤2 s) and VS2 (>2 s). Similarly, V funnel flow time is also classified as two classes, namely VF1 (≤8 s) and VF2 (9 to 25 s). The minimum criteria for passing ability classes by L box ratio is identified as PA1 (≥0.80) and segregation resistance is of two classes, SR1 (15 to 20) and SR2 (≤15).

Corinaldesi and Moriconi [93], when studying SCC with RA and Municipal Solid Waste Ash (MSWA) (as NA substitutes), found that the slump flow is similar and the SF lies in SF3 as per EFNARC standards [92]. Thus, the authors concluded that the NFA and MSWA contribute identically to the SCC’s fluidity. As for the flow time, Corinaldesi and Moriconi [93] obtained identical results with NFA and MSWA and the results lie within the limit.

Grdic et al. [94] obtained that the SF decreased as the RCA integration ratio increased since higher ratios mean more water is absorbed by the RCA. In terms of flow time, Grdic et al. [94] found that it increases with the RCA integration ratio. This is due to the RCA being more angular and having a rougher surface than the CAN.

The addition of silica fume and RP tends to decrease the flowability by absorbing excess free water in the mix [59]. Fineness and shape of the powder particles are two important factors in keeping the initial slump under control [59]. This is also one of the reasons for the addition of SCM in SCC. RP possesses a greater surface area and an irregular microstructure and hence it absorbs more water [33]. RP reduces flow over the time of SCC [33].

Table 10 indicates that the family IA slump flow mostly lies in the class SF2 as per the EFNARC standard [92] and a few in SF1. Similar to family IA, the family IB slump flow lies in the class SF1 and SF2. This indicates that either an increase or a constant level of W/C ratio influences the classification of the slump flow. For family IA, an increase in W/C ratio results in the conversion of slump flow and class SF1 to SF2 is observed but for family IB, an increase in W/C ratio result in a change of slump flow class SF1 and SF2 to SF2. T500 of slump flow from the literature indicates that most of it lies above 2 s, indicating that the flow time lies in class VS2 for both families IA and IB as per EFNARC standard [92]. For family IA, an increase in W/C ratio, resulted in a change of flow time classes from VS1 and VS2 to VS1 but for family IB, an increase in W/C ratio resulted in a change of flow time classes from VS2 to VS1 and VS2.

**Figure 4:**
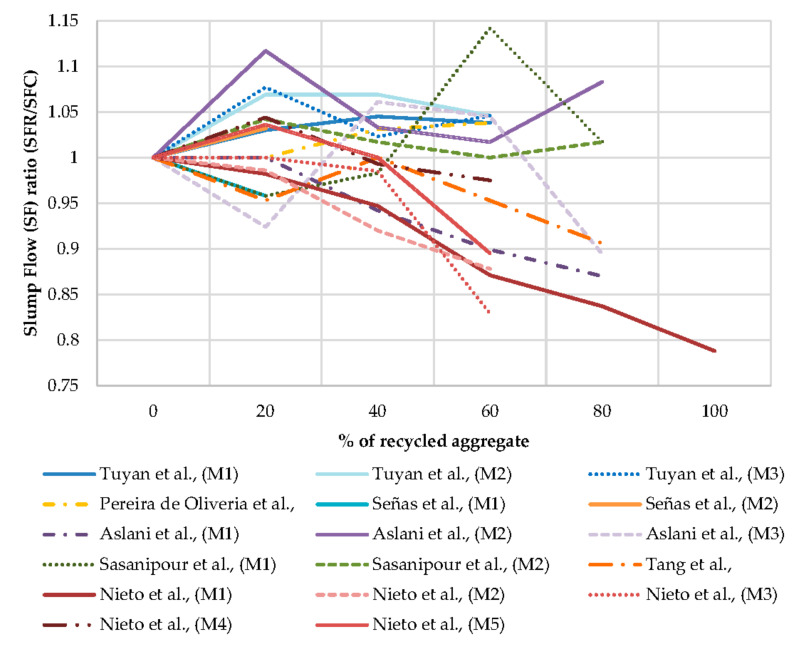
Effect of W/C ratio on slump flow.

From Figure 3 it is observed that most SCCs investigated by previous researchers are lying between SF1 and SF2 as per the EFNARC standard [92]. Most slump flows are lying in the range of 600 mm to 720 mm, indicating much flow for most replacement ratio of coarse aggregate by RA is flowability. From Figure 4 it is observed that the slump flow ratio goes wider with an increase in replacement of NCA by RCA. The slump flow ratio varies by ± 0.05, up to 40% of the replacement of NCA by RA by most researchers is observed.

Señas et al. [49] and Topcu et al. [62], observed that for the same W/C ratio (Family IA), the workability of RCA SCC is decreased and this is also observed in Table 10. Due to the water absorption nature of RCA, the water requirement of the mix is increased to achieve the same workability. Depending upon the quantity and quality of old mortar in RA, the water requirement varies. This is the reason for the lower slump flow for family IA and in most cases, it lies in SF1 as per the EFNARC standard [92]. V funnel time increase with an increase in the W/C ratio is observed for family IA.

Señas et al. [49] found, when fine aggregates are replaced by RFA, there is a need for a greater quantity of admixture to obtain a similar fluidity compared to the reference mixture. This is due to the rough texture and porous nature of RFA.

Usage of fine filler material in a recycled SCC mix fills the pores, which contributes to the greater density, making a homogeneous material and reducing internal bleeding [33]. It is also confirmed by microanalytical studies [95].

Safiuddin et al. [33] observed that an increase in RCA content of the mix results in an increase in V funnel flow time due to RCA possessing greater rugosity and angularity. It is also reported that the increase in slump flow time is directly related to V funnel flow time because RCA possesses a low flowing ability. Passing ability by L box ratio for most of the results in literature lies in class PA1, as per the EFNARC standard [92].

Pereira-de-Oliveira et al. [32] observed that the V-funnel flow time increased for the RCA as replacement ratios like 20% and 40% for NCA, and 40% of the RCA mix had the highest flow time and has the lowest W/C (0.56). All the others have an identical W/C ratio (0.57). Therefore, the authors mention that the V-funnel flow time is powerfully influenced by the W/C ratio.

It is observed that an increase in W/C ratio either in family IA or IB results in the V-funnel flow lying in either class VF1 or VF2. An increase in water content of mix results in increased in-flow time in V-funnel but with the addition of RCA results in a decrease in V-funnel time. The segregation percentage lies below 15% for both families.

Salesa et al. [6] reported that the quantity of cement paste from RA has a greater influence on the water requirement of the mix. An increase in old cement paste increases the quantity of unhydrated cement paste volume, which requires a greater water content to achieve the same workability. The fineness of RCA also affects the water requirement of the mix because the finer particles had a larger surface area to wet.

Pereira-de-Oliveira et al. [32] observed the influence of the W/C ratio and the percentage of SP content on the fresh concrete properties of RCA SCC. A gradual increase in SP dosage is associated with an increase in RCA, it is observed. An increase in RCA in SCC is associated with an increase in the W/C ratio, which can be controlled by the addition of SP. Higher water absorption behavior of RCA results in higher water requirement for lubricating the mixture to flow.

### 4.2. Effect of Water to Powder Ratio on Fresh Concrete Properties

Safiuddin et al. [33] reported that the increase in incorporation ratio results in increased segregation resistance up to lower replacement levels of NCA. For higher replacement levels of NCA, the segregation resistance decreases because the increase in RCA content leads to an increase in the fineness content of the mixture. Grdic et al. [94] found that the increase in RCA content leads to an increase in the W/C ratio, resulting in a decrease in segregation resistance.

From Table 11, the following key observations are made from works of literature:

- An increase in the W/P ratio results in T500 flow time to decrease because the flowability of the mix is better when the suspension of solid materials in liquid media increases;
- Slump flow increases with an increase in W/P ratio;
- SCC flow regarding obstruction (J-ring) increases with an increase in W/P ratio;
- L-Box ratio has no relationship concerning W/P ratio;
- V-funnel initially decreases and then increases to W/P ratio.

**Figure 5:**
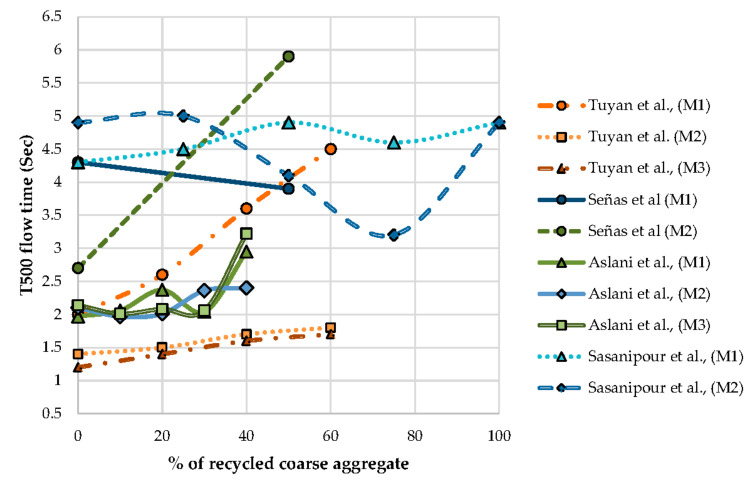
Effect of T500 flow time on the percentage of recycled coarse aggregate.

An increase in replacement percentage of natural aggregate by the coarse aggregate resulting in an increase in flow time is observed in Figure 5.

**Figure 6:**
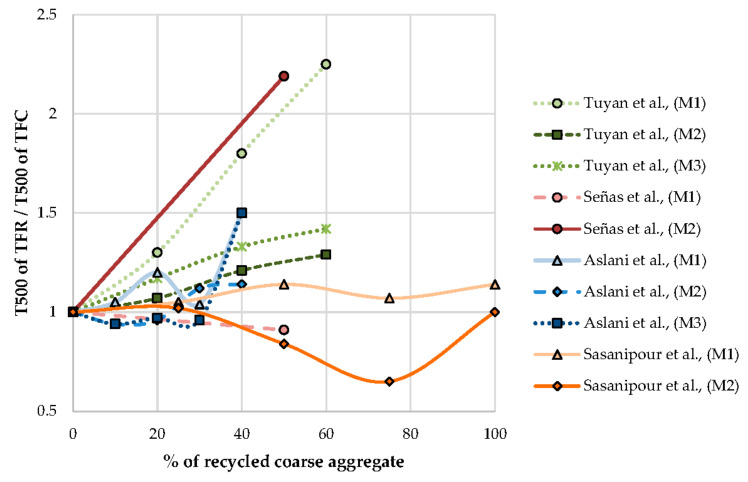
Effect of T500 time flow ratio on the percentage of recycled coarse aggregate.

The time flow ratio is the ratio of T500 time flow of SCC with RCA to SCC with NCA. The time flow ratio increase with an increase in the percentage of recycled content in the SCC mix is observed. To keep the ratio constant, several parameters like the dosage of SP can be adjusted as observed by Aslani et al. [70] From Figure 6, it is observed that up to 40% of the replacement of NCA by RCA results in not much variation in the ratio. The ratio is varied by altering the design parameters, it is observed.

### 4.3. Effect of the Percentage of Superplasticizer on Fresh Concrete Properties

**Figure 7:**
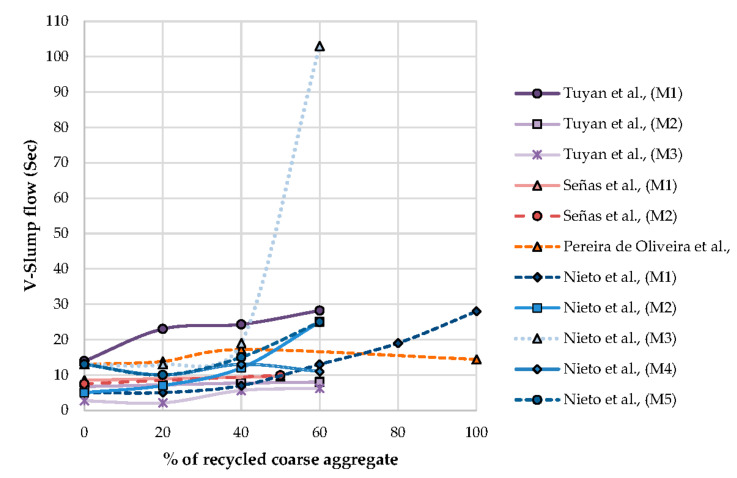
Effect of V-slump flow time on the percentage of recycled coarse aggregate.

Señas et al. [49] observed that by varying percentages and types of SP, the flow parameters lie within the EFNARC standard [92]. Flow class is also not modified when coarse aggregate or fine aggregate is replaced by RA. Admixture with a lower density influences the viscosity with an increase in RA content in mixture, and admixture with a higher density does not influence viscosity, it is observed. This was confirmed by the V-funnel test and it is observed that viscosity nature increases with an increase in RA content. Incorporation of SP to the mixture shows a better presence without any sign of segregation or bleeding. From Figure 7, it is observed that the larger proportions of RA in SCC mixes shows a variation in V-slump flow. Most of the V-slump flow in the range of 5 to 30 sec is observed. An increase with the replacement of NCA by RCA results in an increase in V-slump flow, it is noted.

Salesa et al., [6] 2017 reported that the SCC with RCA has greater flowability compared to conventional SCC and it is influenced by two factors. First, the particle size distribution of RCA and secondly due to the activation of SP from RA apart from SP in the current mix leads to achieving better flowability. The percentage of RCA does not influence air content, as observed by Salesa et al. [6]. Gridic et al. [94] observed that the passing ability lies in the range of 0.94 to 0.98, which increases with increases in the RCA content. Tuyan et al. [14] found that the increase in SP content increases the passing ability of mix with an increase in RCA. Modani and Mohitkar [93] observed that 100% incorporation of RCA content results in a lower passing ability with a higher amount of SP in it. Tuyan et al. [14] reported that the V-funnel flow time increased with an increase in RCA ratio with a lower SP content in it. The inclusion of metakaolin results in a decrease in workability, which is compensated by increasing the percentage of SP [45]. Although there is a decrease in SCC workability, it increases the mechanical strength of SCC [45]. An increase in metakaolin decreases the alkali-silica reaction (ASR) of aggregates in concrete [96]. An increase in Cathode Ray Tunnel Glass (CRTG) in the mixture increases the workability [97].

From Table 12, the following features are observed:

- An increase in the percentage of SP increases in T500 flow time;
- No constant relationship between slump flow and percentage of SP;
- An increase in the percentage of SP increases J-ring flow;
- An increase in the percentage of SP up to 1%, increases bypassing ability, i.e., L-box ratio;
- An increase in the percentage of SP increases segregation resistance.

## 5. Conclusions

The literature results analyzed in this research show that recycled aggregate (RA) can be considered as a feasible alternative material for natural aggregates and is feasible for use in self-compacting concrete (SCC). The fresh properties of SCC with recycled coarse aggregate (RCA) show fully satisfactory results for structural purposes and are classified as per EFNRARC standards. RA would make a significant contribution to sustainability in concrete production, and could directly have an impact on the construction sector and in the circular economy. The following conclusions have been drawn from the development and results of this research study:

- For the family, I mix,
  An increase in the water to cement (W/C) ratio shows a decrease in the percentage of cement content in terms of unit weight.
  An increase in the W/C ratio causes an increase in the supplementary cementitious material (SCM) content of the mixes.
  An increase in the percentage of aggregate in mixes results in the increase in water required to achieve the same workability of the mix.
- For family II mix,
  Increase in water to powder (W/P) ratio, powder content of mix is decreased;
  Increase in W/P ratio, the proportion of fine aggregate content is decreased and coarse aggregate content is increased;
  Increase in W/P ratio, the proportion of replacement of fine aggregate by RFA is decreased.
- For family III mix,
  An increase in the replacement of aggregate by RA increases SP to achieve the same consistency;
  Increases in SP increase the SCM content of the mixture;
  An increase in SP increases the filler quantity of the mix.
- An increase in the W/P ratio results in T500 flow time to decrease because the flowability of the mix is better when the suspension of solid materials in liquid media increases. Slump flow increases with an increase in the W/P ratio. SCC flow about obstruction (J-ring) increases with an increase in the W/P ratio. L-Box ratio has no relationship concerning the W/P ratio. V-funnel initially decreases and then increases to the W/P ratio.
- An increase in the percentage of SP increases in T500 flow time. There is no constant relationship between slump flow and percentage of SP. An increase in the percentage of SP increases J-ring flow. An increase in the percentage of SP up to 1%, increases by passing ability, i.e., L-box ratio. An increase in the percentage of SP increases segregation resistance.

The good results of SCC with RA obtained by some authors should be an incentive to reconsider the use of this type of waste as RA in concrete, which would also help to make the construction process more sustainable. However, more studies would be necessary for its properties as well as the development of standards and guides on the use of RA in concrete.

## Figures and Tables

**Table 1:** Average value for the properties of ingredients other than recycled aggregates used in the literature.

Ingredients	Specific Gravity	Fineness Modulus	Blaine’s Surface Area (kg/cm^2^)	Water Absorption (%)	Bulk Density (kg/m^3^)	References
Cement	3.108		3495.5			Tuyan et al., 2014 [14], Pereira-de-Oliveria et al., 2014 [32], Señas et al., 2016 [49], Duan et al., 2020 [67], Tang et al., 2016 [68], Djelloul et al., 2018 [69], Sasanipour et al., 2019 [56], Aslani et al., 2018 [70], Ouldkhaoua et al., 2020 [45], Nieto et al., 2019 [71], Gupta et al., 2020 [73], Singh et al., 2019 [66], Abed et al., 2020 [74], Xavier et al., 2020 [75], Dapena et al., 2011 [72], Rajhans et al., 2011 [76]
Fine aggregate	2.651	2.76		1.35	1632.5	
Coarse aggregate	2.646	6.05		1.24	1440.25	

**Table 2:** Recycled aggregate properties used in the literature.

Source	Types	AS ^1^ (mm)	FM ^2^ (mm)	CF ^3^ (%)	SG ^4^	W ^5^ (%)	BD ^6^ (kg/m^3^)	References
Waste laboratory made concrete	RCA	-	-	-	2.48	4.80	1410	Tuyan et al., 2014 [14]
Construction and demolition waste	RCA	9.5	5.78	-	-	4.10	1509	Pererira-de-Olivera et al., 2014 [32]
	RCA	19	6.92	-	-	4.05	1485	
Construction work	RCA	12.5	3.77	-	-	-	-	Señas et al., 2016 [49]
Waste infrastructure component	RCA	12	2.59	-	-	6.53	1220	Duan et al., 2020 [67]
Construction and demolition waste facility	RCA	10	-	-	-	7.75	1450	Tang et al., 2016 [68]
Waste from laboratory concrete	RFA	-	3.8	-	2.27	8.87	1258	Djelloul., 2018 [69]
	RCA	9.5	-	-	2.39	7.39	1172	
	RCA	12.5	-	-	2.4	3.21	1154	
Residential and sanitary buildings	RCA	-	-	-	2.36	5.4	-	Sasanipour et al., 2019 [56]
	RFA	-	3.67		2.23	14.8	-	
Demolition waste	RCA	-	-	-	-	5.60	1612	Abed et al., 2020 [74]
Concrete, bricks, asphalt, glass, others, aggregates	RCA	16	-	-	-	6.61	1584	Nieto et al., 2019 [71]
Demolished building	RCA	-	-	-	2.54	2.33	1220	Gupta et al., 2020 [73]
Cathode ray tube funnel glass	RFA	-	2.16	-	2.75	-	-	Ouldkhaoua et al., 2020 [45]

^1^ Aggregate size (mm); ^2^ Fineness modulus; ^3^ Content of fineness (%); ^4^ Specific gravity; ^5^ Water absorption (%); ^6^ Bulk Density (kg/m^3^).

**Table 3:** Average of reference mix proportions (control mix in weight basis) used by several authors from 2010–2020.

Ingredients (Kg/m^3^)	Average Values	Standard Deviation	References
Cement	386.31	83.64	Tuyan et al., 2014 [14], Pereira-de-Oliveria et al. [32], 2014, Señas et al., 2016 [49], Duan et al., 2020 [67], Tang et al., 2016 [68], Djelloul et al., 2018 [69], Sasanipour et al., 2019 [56], Aslani et al., 2018 [70], Ouldkhaoua et al., 2020 [45], Nieto et al., 2019 [71], Gupta et al., 2020 [73], Singh et al., 2019 [66], Abed et al., 2020 [74], Xavier et al., 2020 [75], Dapena et al., 2011 [72], Rajhans et al., 2011 [76]
SCM	191.4	80.84	
Filler	166.5	182.37	
Fine aggregate	823.95	214.93	
Coarse Aggregate	749.26	113.77	
Water	194.07	23.52	
SP	3.62	1.67	
Unit weight	2335.61	46.12	

**Table 4:** Mix proportion (weight basis) adopted from Tuyan et al., 2014 [14], Pereira-de-Oliveria et al., 2014 [32], Señas et al., 2016 [49] and Duan et al., 2020 [67].

RCA Content (%)	FRA Content (%)	Cement (kg/m^3^)	W/C ^1^	% SP ^2^	Other Waste	Unit Weight (kg/m^3^)	W/B ^3^	W/P ^4^	References
0	-	315	0.62	1.55	Fly ash	2294	0.42	0.32	Tuyan et al., 2014 [14]
0	-	315	0.69	1.04		2260	0.48	0.36	
0	-	315	0.76	0.69		2224	0.53	0.40	
20	-	315	0.62	1.74		2295	0.42	0.32	
20	-	315	0.69	1.07		2260	0.48	0.36	
20	-	315	0.76	0.76		2224	0.53	0.40	
40	-	315	0.62	1.18		2295	0.42	0.32	
40	-	315	0.69	1.11		2260	0.48	0.36	
40	-	315	0.76	0.85		2225	0.53	0.40	
60	-	315	0.62	1.96		2295	0.42	0.32	
60	-	315	0.69	1.17		2260	0.48	0.36	
60	-	315	0.76	0.95		2225	0.53	0.40	
0	-	284.9	0.57	1.19	-	2358.5	0.25	0.129	Pereira-de-Oliveria et al., 2014 [32]
20	-	284.9	0.57	1.68		2349.8	0.25	0.130	
40	-	284.9	0.56	1.61		2335.3	0.25	0.128	
100	-	284.9	0.56	2.1		2282.2	0.25	0.130	
0	0	332	0.4	1.25		2400	0.4	0.5	Señas et al., 2016 [49]
50	0	332	0.4	1.50		2359	0.4	0.5	
50	20	332	0.4	1.75		2341	0.4	0.5	
0	0	332	0.4	0.87		2398	0.4	0.5	
50	0	332	0.4	1.62		2359	0.4	0.5	
50	20	332	0.4	1.87		2342	0.4	0.5	
0	0	430.5	0.57	0.355	Fly ash Silica fume	2301.9	0.4	0.4	Duan et al., 2020 [67]
0	10	430.5	0.57	0.355		2301.9	0.44	0.4	
25	10	430.5	0.57	0.355		2301.9	0.44	0.4	
50	10	430.5	0.57	0.355		2301.9	0.44	0.4	
100	10	430.5	0.57	0.355		2301.9	0.44	0.4	
0	20	430.5	0.57	0.355		2301.9	0.50	0.4	
25	20	430.5	0.57	0.355		2301.9	0.50	0.4	
50	20	430.5	0.57	0.355		2301.9	0.50	0.4	
100	20	430.5	0.57	0.355		2301.9	0.50	0.4	

^1^ water/cement ratio; ^2^ % of superplasticizer to cement; ^3^ water/binder ratio; ^4^ water/powder ratio.

**Table 5:** Mix proportion adopted from Tang et al., 2020 [68], Djelloul et al., 2018 [69], Sasanipour et al., 2019 [56].

RCA Content (%)	FRA Content (%)	Cement (kg/m^3^)	W/C ^1^	% SP ^2^	Other Waste	Unit Weight (kg/m^3^)	W/B ^3^	W/P ^4^	References
0	-	445	0.49	1.011	Fly ash Silica fume	2329.5	0.35	0.35	Tang et al., 2020 [68]
25	-	445	0.49	1.011		2316.5	0.35	0.35	
50	-	445	0.49	1.011		2304.5	0.35	0.35	
75	-	445	0.49	1.011		2292.5	0.35	0.35	
100	-	445	0.49	1.011		2279.5	0.35	0.35	
0	0	507	0.37	1.5	GGBFS	2352	0.37	0.38	Djelloul et al., 2018 [69]
25	25	507	0.37	1.5		2307	0.37	0.38	
50	50	507	0.37	1.5		2261	0.37	0.38	
75	75	507	0.37	1.5		2265	0.37	0.38	
100	100	507	0.37	1.5		2170	0.37	0.38	
0	0	434	0.37	1.5		2353	0.37	0.38	
25	25	434	0.37	1.5		2307	0.37	0.38	
50	50	434	0.37	1.5		2261	0.37	0.38	
75	75	434	0.37	1.5		2265	0.37	0.38	
100	100	434	0.37	1.5		2170	0.37	0.38	
0	0	359	0.37	1.5		2353	0.37	0.38	
25	25	359	0.37	1.5		2307	0.37	0.38	
50	50	359	0.37	1.5		2261	0.37	0.38	
75	75	359	0.37	1.5		2265	0.37	0.38	
100	100	359	0.37	1.5		2170	0.37	0.38	
0	0	420	0.4	0.9	Silica fume	2322	0.4	0.28	Sasanipour et al., 2019 [56]
0	0	386.4	0.4	1		2322	0.4	0.28	
25	0	420	0.4	1.06		2317	0.4	0.28	
50	0	420	0.4	1.09		2294	0.4	0.28	
75	0	420	0.4	1.05		2287	0.4	0.28	
100	0	420	0.4	1		2274	0.4	0.28	
25	0	386.4	0.4	1		2317	0.4	0.28	
50	0	386.4	0.4	1.09		2299	0.4	0.28	
75	0	386.4	0.4	1		2287	0.4	0.28	
100	0	386.4	0.4	1		2274	0.4	0.28	
0	25	420	0.4	0.92		2287	0.4	0.28	
0	25	420	0.4	0.9		2320	0.4	0.28	

^1^ water/cement ratio; ^2^ % of SP to cement; ^3^ water/binder ratio; ^4^ water/powder ratio.

**Table 6:** Mix proportion adopted from Aslani et al., 2018 [71], Ouldkhaoua et al., 2020 [46], Nieto et al., 2019 [72], Dapena et al., 2011 [73].

RCA Content (%)	FRA Content (%)	Cement (kg/m^3^)	W/C ^1^	% SP ^2^	Other Waste	Unit Weight (kg/m^3^)	W/B ^3^	W/P ^4^	References
0	0	180	1.125	1.667	Fly ash GGBFS Silica fume Crumbled rubber Scoria	2332	0.45	0.25	Aslani et al., 2018 [70]
10	10	180	1.125	1.222		2316	0.45	0.26	
20	20	180	1.125	1.333		2301	0.45	0.27	
30	30	180	1.125	1.444		2286	0.45	0.29	
40	40	180	1.125	1.556		2270	0.45	0.30	
0	0	180	1.125	1.333		2245	0.45	0.25	
0	10	180	1.125	1.472		2238	0.45	0.26	
0	20	180	1.125	1.556		2231	0.45	0.27	
0	30	180	1.125	1.667		2223	0.45	0.29	
0	40	180	1.125	1.778		2215	0.45	0.30	
0	0	180	1.125	1.111		2168	0.45	0.25	
0	10	180	1.125	1.222		2160	0.45	0.26	
0	20	180	1.125	1.333		2152	0.45	0.27	
0	30	180	1.125	1.444		2144	0.45	0.29	
0	40	180	1.125	1.556		2136	0.45	0.30	
0	0	446	0.4	0.8	MK	2380	0.4	0.4	Ouldkhaoua et al., 2020 [45]
0	0	446	0.42	0.85	CRTG	2380	0.4	0.4	
0	10	446	0.42	0.85		2380	0.4	0.4	
0	20	446	0.42	0.85		2380	0.4	0.4	
0	30	446	0.42	0.83		2380	0.4	0.4	
0	40	446	0.42	0.83		2380	0.4	0.4	
0	50	446	0.42	0.8		2380	0.4	0.4	
0	0	422	0.44	1.1		2380	0.4	0.41	
0	10	422	0.44	1.1		2380	0.4	0.41	
0	20	422	0.44	1.1		2380	0.4	0.41	
0	30	422	0.44	1.05		2380	0.4	0.41	
0	40	422	0.44	1		2380	0.4	0.41	
0	50	422	0.44	0.95		2380	0.4	0.41	
0	0	399	0.47	1.2		2380	0.4	0.41	
0	10	399	0.47	1.2		2380	0.4	0.41	
0	20	399	0.47	1.2		2380	0.4	0.41	
0	30	399	0.47	1.15		2380	0.4	0.41	
0	40	399	0.47	1.15		2380	0.4	0.41	
0	50	399	0.47	1.1		2380	0.4	0.41	
0	-	367	0.55	1.5	-	2354	0.55	0.42	Nieto et al., 2019 [71]
20	-	367	0.55	1.5		2349	0.55	0.42	
40	-	367	0.55	1.5		2344	0.55	0.42	
60	-	367	0.55	1.5		2339	0.55	0.42	
80	-	367	0.55	1.5		2334	0.55	0.42	
100	-	367	0.55	1.5		2329	0.55	0.42	
0	-	386	0.5	1.5		2371	0.5	0.38	
20	-	386	0.5	1.5		2366	0.5	0.38	
40	-	386	0.5	1.5		2361	0.5	0.38	
60	-	386	0.5	1.5		2356	0.5	0.38	
0	-	408	0.45	1.5		2390	0.45	0.35	
20	-	408	0.45	1.5		2385	0.45	0.35	
40	-	408	0.45	1.5		2380	0.45	0.35	
60	-	408	0.45	1.5		2375	0.45	0.35	
0	-	408	0.45	1.5		2390	0.45	0.35	
20	-	408	0.45	1.5		2391	0.45	0.36	
40	-	408	0.45	1.5		2391	0.45	0.37	
60	-	408	0.45	1.5		2392	0.45	0.38	
0	-	408	0.45	1.5		2390	0.45	0.35	
20	-	408	0.45	1.5		2391	0.45	0.36	
40	-	408	0.45	1.5		2391	0.45	0.37	
60	-	408	0.45	1.5		2392	0.45	0.38	
0	0	380	0.5	0.7	-	2290	0.5	0.5	Dapena et al., 2011 [72]
20	0	380	0.5	0.7		2330	0.5	0.5	
20	5	380	0.5	0.7		2263	0.5	0.5	
20	10	380	0.5	0.7		2263	0.5	0.5	
50	0	380	0.5	0.7		2224	0.5	0.5	
50	5	380	0.5	0.7		2224	0.5	0.5	
50	10	380	0.5	0.7		2224	0.5	0.5	
100	0	380	0.5	0.7		2161	0.5	0.5	
100	5	380	0.5	0.7		2161	0.5	0.5	
100	10	380	0.5	0.7		2161	0.5	0.5	

^1^ water/cement ratio; ^2^ % of SP to cement; ^3^ water/binder ratio; ^4^ water/powder ratio.

**Table 7:** Grouping of mixed design concerning water-cement ratio (design parameters) based on the weight of ingredients (all ingredients are in terms of percentage).

Fª	W/C	C (%)	SCM (%)	Filler (%)	FA (%)	CA (%)	RFA (%)	RCA (%)	SP (%)	W (%)	References
IA	0.42	18.67	0.79	-	34.27–19.04	34.32	3.81–19.03	-	0.17	7.87	Ouldkhaoua et al., 2020 [45]
	0.44	17.71	1.57	-	34.32–19.10	34.50	3.81–19.07	-	0.18–0.21	7.88	Ouldkhaoua et al., 2020 [45]
	0.45	17.18–17.11	-	5.18–5.16	40.0–39.83	24.42–12.21	-	17.68–0	0.26	7.75–7.71	Nieto et al., 2019 [71]
	0.47	16.76	2.35	-	34.38–19.07		3.82–19.10	-	0.21–0.23	7.89	Ouldkhaoua et al., 2020 [45]
	0.50	16.38–16.31	-	4.92–4.90	40.32–40.15	24.51–12.31	-	17.83–0	0.25	8.19–8.16	Nieto et al., 2019 [71]
	0.55	15.76–15.62	-	4.72–4.68	40.79–40.44	24.69–0	-	30.06–0	0.24	8.67–8.60	Nieto et al., 2019 [71]
	0.62	13.73	5.85	7.06	32.17	25.9712.98	-	19.47–6.49	0.27–0.24	8.45	Tuyan et al., 2014 [14]
	0.69	13.93	5.97	6.95	31.55	24.56 12.74	-	19.12–6.37	0.16–0.15	9.56	Tuyan et al., 2014 [14]
	0.76	14.16	6.07	6.79	30.92	24.94–12.49	-	18.70–6.25	0.13–0.11	10.71	Tuyan et al., 2014 [14]
IB	0.4	13.89–14.18	-	3.54	45.15–36.17	15.89–16.01	8.33	14.11–14.22	0.21–0.26	7.09–7.04	Señas et al., 2016 [49]
	0.49	19.21–19.52	7.99–8.12	-	35.18–35.75	0–21.37		0–26.33	0.20–0.19	9.50–9.65	Tang et al., 2016 [68]
	0.56	12.20–12.48	15.85–16.22	25.59–26.19	5.70–5.84	20.76		12.82–31.90	0.20–0.26	6.88–7.12	Pereira-de-Oliveria et al., 2014 [32]
	0.57	12.12	15.75	25.43	5.67	27.50		6.37	0.19–0.26	6.94	Pereira-de-Oliveria et al., 2014 [32]
	0.57	18.70	2.67–5.34	2.6–5.34	28.67	16.94–25.41	-	8.47–33.89	0.07	28.67	Duan et al., 2020 [67]

Fª = Family; W/C = Water/cement ratio; C = Cement; SCM = Supplementary cementitious materials: FA = Fine aggregate; CA = Coarse aggregate; RFA = Recycled fine aggregate; RCA = Recycled coarse aggregate; W = Water.

**Table 8:** Grouping of mix design concerning water to powder ratio (design parameters) based on the weight of ingredients (all ingredients are in terms of percentage).

Fª	W/C	C (%)	SCM (%)	Filler (%)	FA (%)	CA (%)	RFA (%)	RCA (%)	SP (%)	W (%)	References
II	0.28	18.12–16.99	9.04–7.42	-	45.39–44.54	23.00–5.75	9.75–9.62	21.02–5.16	0.20–0.16	7.38–7.25	Sasanipour et al., 2019 [56]
	0.32	13.73	5.85	7.06	32.17	25.97–12.98	-	19.47–6.49	0.27–0.24	8.45	Tuyan et al., 2014 [14]
	0.35	17.18–17.11	-	5.18–5.16	40.00–39.83	24.42–12.21	-	17.68–0	0.26	7.75–7.71	Nieto et al., 2019 [71]
	0.35	19.21–19.52	7.99–8.12	-	35.18–35.75	0–21.37		0–26.33	0.20–0.19	9.50–9.65	Tang et al., 2016 [68]
	0.36	13.93	5.97	6.95	31.55	24.56–12.74	-	19.12–6.37	0.16–0.15	9.56	Tuyan et al., 2014 [14]
	0.38	16.38–16.31	-	4.92–4.90	40.32–40.15	24.51–12.31	-	17.83–0	0.25	8.19–8.16	Nieto et al., 2019 [71]
	0.38	18.81–16.54	6.82–3.16	-	29.58–0	23.90–0	37.56–8.83	29.82–11.17	0.33–0.35	8.89–8.36	Djelloul et al., 2018 [69]
	0.40	18.70	2.67–5.34	2.67–5.34	28.67	16.94–25.41	-	8.47–33.89	0.07	28.67	Duan et al., 2020 [67]
	0.40	14.16	6.07	6.79	30.92	24.94–12.49	-	18.70–6.25	0.13–0.11	10.71	Tuyan et al., 2014 [14]
	0.41	18.67–16.76	2.35–0.79	-	34.38–19.04	34.50–34.32	19.10–3.81	-	0.23–0.17	7.89–7.87	Ouldkhaoua et al., 2020 [45]
	0.42	15.76–15.62	-	4.72–4.68	40.79–40.44	24.69–0	-	30.06–0	0.24	8.67–8.60	Nieto et al., 2019 [71]
	0.50	13.89–14.18	-	3.54	45.15–36.17	15.89–16.01	8.33	14.11–14.22	0.21–0.26	7.09–7.04	Señas et al., 2016 [49]
	0.50	16.79–16.30	-	-	32.89–31.94	33.44–32.48	0.83–0.42	8.12–7.52	0.12–0.11	8.39–8.15	Dapena et al., 2011 [72]

Fª = Family; W/C = Water/cement ratio; C = Cement; SCM = Supplementary cementitious materials; FA = Fine aggregate; CA = Coarse aggregate; RFA = Recycled fine aggregate; RCA = Recycled coarse aggregate; W = Water.

**Table 9:** Grouping of mix design to a percentage of superplasticizer (design parameters) based on the weight of ingredients (all ingredients are in terms of percentage).

Fª	SP (%)	C (%)	SCM (%)	Filler (%)	FA (%)	CA (%)	RFA (%)	RCA (%)	W (%)	References
III	0.35	18.70	2.67–5.34	2.67–5.34	28.67	16.94–25.41	-	8.47–33.89	28.67	Duan et al., 2020 [67]
	0.8–1.2	18.67–16.76	2.35–0.79	-	34.38–19.04	34.50–34.32	19.10–3.81	-	7.89–7.87	Ouldkhaoua et al., 2020 [45]
	0.9–1.09	18.12–16.99	9.04–7.42	-	45.39–44.54	23.00–5.75	9.75–9.62	21.02–5.16	7.38–7.25	Sasanipour et al., 2019 [56]
	0.95–0.76	14.16	6.07	6.79	30.92	24.94–12.49	-	18.70–6.25	10.71	Tuyan et al., 2014 [14]
	1.011	19.21–19.52	7.99–8.12	-	35.18–35.75	0–21.37		0–26.33	9.50–9.65	Tang et al., 2016 [68]
	1.18–1.08	13.93	5.97	6.95	31.55	24.56–12.74	-	19.12–6.37	9.56	Tuyan et al., 2014 [14]
	1.5	15.76–15.62	-	4.72–4.68	40.79–40.44	24.69–0	-	30.06–0	8.67–8.60	Nieto et al., 2019 [71]
	1.5	16.38–16.31	-	4.92–4.90	40.32–40.15	24.51–12.31	-	17.83–0	8.19–8.16	Nieto et al., 2019 [71]
	1.5	17.18–17.11	-	5.18–5.16	40.00–39.83	24.42–12.21	-	17.68–0	7.75–7.71	Nieto et al., 2019 [71]
	1.5	18.81–16.54	6.82–3.16	-	29.58–0	23.90–0	37.56–8.83	29.82–11.17	8.89–8.36	Djelloul et al., 2018 [69]
	1.5–1.88	13.89–14.18	-	3.54	45.15–36.17	15.89–16.01	8.33	14.11–14.22	7.09–7.04	Señas et al., 2016 [49]
	1.97–1.75	13.73	5.85	7.06	32.17	25.97–12.98	-	19.47–6.49	8.45	Tuyan et al., 2014 [14]
	2.11–1.19	12.48–12.12	16.22–15.75	26.18–25.43	5.83–5.67	27.50–0	-	31.89–6.37	7.12–6.95	Pereira-de-Oliveria et al., 2014 [32]
	2.66	16.79–16.30	-	-	32.89–31.94	33.44–32.48	0.83–0.42	8.12–7.52	8.39–8.15	Dapena et al., 2011 [72]

Fª = Family; W/C = Water/cement ratio; C = Cement; SCM = Supplementary cementitious materials; FA = Fine aggregate; CA = Coarse aggregate; RFA = Recycled fine aggregate; RCA = Recycled coarse aggregate; W = Water.

**Table 10:** Classification of the family I as per EFNARC standards [92] and the effect of the W/C ratio on fresh concrete properties.

Fª	W/C	T500SF (s)	SF (mm)	T500 J-Ring (s)	d_max_ J-Ring (mm)	L-Box H2/H1	V-f (s)	Sieve Seggreg. (%)	References
IA	0.42	-	-	-	-	0.87–0.97 (PA1)	6.1–8.6 (VF1–VF2)	0–15 (SR2)	[45]
	0.44	-	-	-	-	0.84–0.95 (PA1)	6.2–9 (VF1–VF2)	6–14 (SR2)	[45]
	0.45	2–6 (VS1–VS2)	568–715 (SF1)	10–248	578–675	-	5–103 (VF1–VF2)	-	[71]
	0.47	-	-	-	-	0.81–0.91 (PA1)	9.2–10 (VF2)	5–13 (SR2)	[45]
	0.50	2–5 (VS1–VS2)	628–715 (SF2)	2–41	445–683	-	5–25 (VF1–VF2)	-	[71]
	0.55	1–5 (VS1–VS2)	570–723 (SF1–SF2)	2–40	590–720	-	5–28 (VF1–VF2)	-	[71]
	0.62	2–4.6 (VS1–VS2)	665–690 (SF2)	-	-	0.89–0.95 (PA1)	13.9–28.2 (VF2)	-	[14]
	0.69	1.4–1.8 (VS1)	655–700 (SF2)	-	-	0.81–0.90 (PA1)	6.7–8.2 (VF1–VF2)	-	[14]
	0.76	1.2–1.7 (VS1)	650–700 (SF2)	-	-	0.60–0.7	2.7–6.2 (VF1)	-	[14]
IB	0.4	2.7–10.5 (VS2)	610–710 (SF1–SF2)	6.1–13.8	570–670	-	7.5–14 (VF1-VF2)	-	[49]
	0.49	2.9–4.3 (VS2)	700–720 (SF2)	-	-	0.80–0.89 (PA1)	-	5.20–9.90 (SR2)	[68]
	0.56	-	670 (SF2)	-	-	-	17.2 (VF2)	-	[32]
	0.57	1.8–3.4 (VS1–VS2)	660–750 (SF2)	-	-	0.78–0.92 (PA1)	4.1–6.1 (VF1)	-	[67]
	0.57	-	650–675 (SF2)	-	-	-	13–14.4 (VF2)	-	[32]

Fª = Family; W/P = Water/power ratio; SF = Slump Flow; V-f = V-funnel.

**Table 11:** Classification of family II as per EFNARC standard [92] and the effect of the W/P ratio on fresh concrete properties.

Fª	W/P	T500 SF(s)	SF (mm)	T500 J-Ring (s)	dmax J-Ring (mm)	L-Box H2/H1	V-f (s)	Sieve Segregation (%)	References
II	0.28	3.1–7.1 (VS2)	570–670 (SF1–SF2)	4–11	545–656	0.87–0.97 (PA1)	-	-	[56]
	0.32	2–4.5 (VS2)	665–695 (SF2)	-	-	0.84–0.95 (PA1)	2.3–28.2 (VF1–VF2)	-	[68]
	0.35	2–15 (VS2)	560–685 (SF1–SF2)	5–8	643–675	-	7–25 (VF1–VF2)		[71]
	0.35	2.9–4.3 (VS2)	700–720 (SF2)	-	-	0.81–0.91 (PA1)	-	5.20–9.90 (SR2)	[68]
	0.36	1.4–1.8 (VS1)	655–700 (SF1–SF2)	-	-	-	2.7–8 (VF1)	-	[68]
	0.38	1–6 (VS1–VS2)	613–715 (SF1–SF2)	2–41	365–683	-	5–25 (VF1–VF2)	-	[71]
	0.38	2.9–4.9 (VS2)	713–792 (SF2–SF3)	-	-	0.89–0.95 (PA1)	6.16–23.20 (VF1–VF2)	5.10–16.50 (SR1–SR2)	[69]
	0.40	1.8–3.4 (VS1–VS2)	640–750 (SF1–SF2)	-	-	0.81–0.90 (PA1)	4.1–6.1 (VF1)	-	[67]
	0.40	1.2–1.7 (VS1)	650–700 (SF1–SF2)	-	-	0.60–0.7	2.7–7.2 (VF1)	-	[67]
	0.41	-	-	-	-	-	6.5–9 (VF1–VF2)	6–14 (SR2)	[45]
	0.42	1–6 (VS1–VS2)	570–723 (SF1–SF2)	2–40	475–720	0.80–0.89 (PA1)	5–28 (VF1–VF2)	-	[71]
	0.50	2.7–10.5 (VS2)	610–710 (SF1–SF2)	6.1–13.8	570–670	-	7.5–14 (VF1–VF2)	-	[49]

Fª = Family; W/P = Water/power ratio; SF = Slump Flow; V-f = V-funnel.

**Table 12:** Classification of family III as per EFNARC standard [95] and effect of % of SP on fresh concrete properties.

Fª	SP (%)	T500 SF(s)	SF (mm)	T500 J-Ring (s)	d_max_ J-Ring (mm)	L-Box H2/H1	V-f (s)	Sieve Seggreg. (%)	References
III	0.35	1.8–3.4 (VS1–VS2)	640–750 (SF1–SF2)	-	-	0.78–0.925	4.1–6.1 (VF1)	-	[67]
	0.8–1.2	-	-	-	-	0.87–0.99 (PA1)	6.1–8.6 (VF1–VF2)	7–15 (SR2)	[45]
		-	-	-	-	0.84–0.95 (PA1)	6.2–9 (VF1–VF2)	6–14 (SR2)	[45]
		-	-	-	-	0.81–0.91 (PA1)	9.2–10 (VF2)	5–13 (SR2)	[45]
	0.9–1.09	3.1–7.1 (VS2)	570–685 (SF1–SF2)	4–11	545–656	-	-	-	[56]
	0.9–0.76	1.2–1.7 (VS1)	650–700 (SF1–SF2)	-	-	0.60–0.70 (PA1)	2.7–6.2 (VF1)	-	[68]
	1.01	2.9–4.3 (VS2)	700–720 (SF2)	-	-	0.80–0.89 (PA1)	-	5.20–9.90 (SR2)	[68]
	1.18–1.08	1.4–1.8 (VS1)	665–700 (SF2)	-	-	0.89–0.90 (PA1)	7.2–13.9 (VF1–VF2)	-	[68]
	1.5	1–6 (VS1–VS2)	570–723 (SF1–SF2)	2–40	475–720	-	5–28 (VF1–VF2)	-	[71]
	1.5	1–5 (VS1–VS2)	628–715 (SF1–SF2)	2–41	445–683	-	5–25 (VF1–VF2)	-	[71]
	1.5	2–15 (VS2)	568–685 (SF1–SF2)	5–8	353–675	-	13–103 (VF2)	-	[71]
	1.5	2.9–4.9 (VS2)	713–792 (SF2)	-	-	0.75–1 (PA1)	6.16–23.20 (VF1–VF2)	5.10–16.50 (SR1–SR2)	[69]
	1.55–1.88	2.7–10.5 (VS2)	610–710 (SF1–SF2)	6.1–13.8	570–660	-	8.5–14 (VF2)	-	[49]
	1.97–1.75	2.6–4.5 (VS2)	685–695 (SF2)	-	-	0.95–0.95 (PA1)	23–28.2 (VF2)	-	[68]
	2.11–1.19	-	650–675 (SF1–SF2)	-	-	-	13–14.4 (VF2)	-	[32]

Fª = Family; W/P = Water/power ratio; SF = Slump Flow; V-f = V-funnel.
